# Identification of Aspartyl-tRNA Synthetase as Co-purifying with Wheat Germ eIF2

**DOI:** 10.17912/micropub.biology.001594

**Published:** 2025-07-09

**Authors:** Ophelia Papoulas, Edward M. Marcotte, Karen S. Browning

**Affiliations:** 1 Department of Molecular Biosciences, The University of Texas at Austin, Austin, TX 78712 USA

## Abstract

Eukaryotic translation initiation factor 2 (eIF2) is among the best-studied of the translation initiation factors, but early preparations from wheat germ consistently showed a co-purifying protein of ~61kDa. As this protein was never identified, we revisited the question of its identity using mass spectrometry on an archived Coomassie-stained and dried gel of eIF2 purified in 1991. The co-purifying protein, aspartyl-tRNA synthetase, is notable for serving as a receptor for the R enantiomer of β-aminobutyric acid, with links to stress-induced eIF2α phosphorylation, highlighting the potential for stable “super complexes” connecting translation initiation with stress responses.

**Figure 1. Identification of co-purifying protein with wheat germ eIF2:**
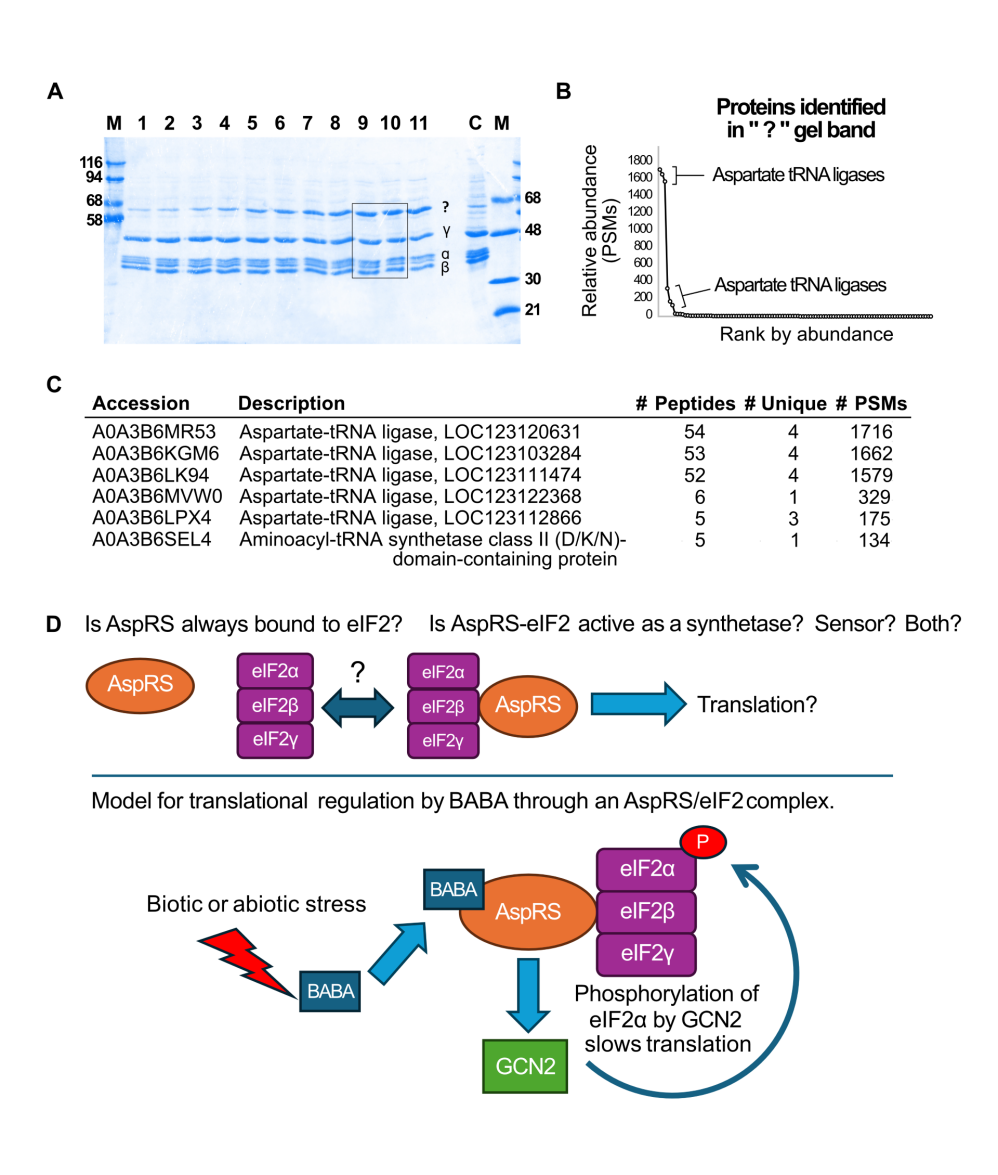
A. Dried SDS PAGE gel used to identify the mystery protein. Lanes 1-11 represent fractions from a phosphocellulose column eluted with a 250-500mM KCl gradient as described (Lax *, et al.* , 1986). M are marker lanes as indicated, and C is a control of a previous eIF2 preparation. B. Relative abundance by number of peptide spectral matches (PSMs) of the 106 proteins identified in the “?” band. C. Table of the six most abundant proteins identified. Because wheat is hexaploid, these six entries represent highly related gene products. Headers indicate Uniprot accessions and descriptions, total and unique peptides identified, and total peptide spectral matches per protein. D. Remaining questions about the association of Asp-tRNA synthetase with the eIF2 complex and a hypothetical model for its potential role in the regulation of eIF2/translation initiation by BABA.

## Description

eIF2 is among the most studied of the translation initiation factors, first purified from rabbit reticulocytes (Merrick *et al.* , 1975) and subsequently from wheat germ (Benne *et al.* , 1980, Clarke *et al.* , 1987, Spremulli *et al.* , 1979). However, in these early preparations of wheat germ eIF2, a protein of ~61kDa was often obtained along with the expected 3 subunits of eIF2, α, β and γ (Benne *, et al.* , 1980, Clarke *, et al.* , 1987, Spremulli *, et al.* , 1979). Further purification methods indicated that the contaminant could be removed using tight salt gradients on ion-exchange resins to remove the contaminant and retain the expected 3 subunits (Treadwell *et al.* , 1979, Walthall *et al.* , 1979). However, the co-purification of this ~61 kDa protein through ammonium sulfate precipitation, anionic and cationic exchange suggests that it is tightly associated with the complex and potentially has some function with eIF2. The recent work by McWhite et al (2020) explored the deep conservation of protein complexes in plants including a conserved tRNA-multi-synthetase complex (McWhite *et al.* , 2020). This work renewed an interest by one of the authors (KSB) in determining what the contaminant might be in relation to the role of eIF2 in initiation of translation. A Coomassie-stained and dried gel of eIF2 purified in 1991 (shown in Figure 1A ) was used to identify the mystery protein.

As shown in Figure 1B- C, mass spectrometry revealed the expected 3 subunits of eIF2α, β, γ and the mystery contaminant to be aspartyl-tRNA synthetase (AspRS). Interestingly, in *Arabidopsis* aspartyl-tRNA synthetase (IBI1, AT4G31180) functions as a receptor for enantiomer specific R-BABA (β-aminobutyric acid) (Schwarzenbacher *et al.* , 2014). R-BABA primes the plant immune system to respond to various pathogens leading to accumulation of aspartic acid and results in phosphorylation of eIF2α by GCN2 due to increased amounts of uncharged tRNA (Luna *et al.* , 2014). However, although phosphorylation by GCN2 regulates reduced growth regulation by BABA, it does not appear to affect the immune response suggesting that there are at least two pathways of regulation by BABA (Luna *, et al.* , 2014). Further studies have shown that IBI1 interacts with VOZ1 and VOZ2 transcription factors and has a role in abscisic acid (ABA) signaling in pathogen response (Schwarzenbacher *et al.* , 2020). A rice thermo-sensitive mutant (ylc3) with reduced levels of chlorophyll, altered chloroplasts and increased aspartate, asparagine and glutamine levels was identified as aspartyl-tRNA synthetase. This mutant was also shown to have increased levels of eIF-2α phosphorylation due to the increase of uncharged aspartyl-tRNA (Liu *et al.* , 2022).

The identification of aspartyl-tRNA synthetase that co-purifies with eIF2 and functions as a receptor for a signaling molecule suggests that in the cell there is an intimate relationship with the regulation of translation in response to pathogens and other biotic and abiotic stresses. Although the first part of this mystery is solved, it now has opened up many more questions about how “super complexes” are formed and regulated within cells. As shown in Figure 1D, it is unknown at this time whether the aspartyl-tRNA synthetase has a dual role both as a receptor to sense the presence of R-BABA and to regulate the activity of eIF2 though phosphorylation by GCN2. It is also unknown if aspartyl tRNA synthetase bound to eIF2 functions as a synthetase or is also present in the tRNA-multi-synthetase complex. It remains to be determined whether there are multiple separate functions of aspartyl-tRNA synthetase and if there are further relevant interactions of these complexes within the cell yet to be discovered.

## Methods

**Sample processing.** Wheat germ eIF2 was purified as described and resolved by SDS PAGE (Lax *et al.* , 1986). Starting with a dried Coomassie stained gel from 1991, the two lanes (See Fig. 1A ) were cut and soaked as follows based on the method of Murphy et al (Murphy *et al.* , 2018) to rehydrate and release the gel from the paper backing. The gel segment was soaked sequentially in 30% methanol/5% acetic acid/5% glycerol, 5% glycerol/1% acetic acid, 1% glycerol/1% acetic acid, and finally dH _2_ 0, to allow removal of paper backing. Each band of interest was then excised, diced, and transferred to a low protein binding Eppendorf tube to be processed using a standard in-gel trypsin digest method (Goodman *et al.* , 2018). Trypsin-digested peptides were extracted from the gel, centrifuged 14,000 x g 10 min to remove residual gel pieces and paper fibers, then desalted using Thermo Scientific Hypersep Spin Tip C-18 (60109-412), dried, and resuspended in 5% acetonitrile/0.1% formic acid for LC-MS-MS.

**Mass Spectrometry.** Spectra were collected on a Thermo Orbitrap Fusion Lumos Tribrid Mass Spectrometer with a trap to column configuration (Thermo Scientific Acclaim PepMap 100 # 64535, EASY-Spray C18 Reversed Phase HPLC Column #ES902) using a data-dependent 75 minute top speed collection method with a 60 minute 3-40% acetonitrile gradient, dynamic exclusion after one observation for 15 seconds, and stepped HCD (27/30/33). RAW files were individually processed in Proteome Discoverer 2.5 using the PWF Tribrid_Basic_fixed_valuePSM_SequestHT workflow with the following settings: trypsin digestion with up to 2 missed cleavages, static carbamidomethyl modification of cysteine, and dynamic modifications of oxidized methionine, and protein N-termini as Met-loss, acetylation, or Met-loss + acetylation. Spectra were searched against the Uniprot *Triticum aestivum* fasta (UP000019116_4565) and a standard contaminants file. Resulting msf files from all samples were reprocessed to create a single output table using the CWF_Basic_pdConsensusWF workflow with a 1% peptide level and 1% protein level FDR and strict parsimony for protein grouping. Data are available from the MassIVE data repository under accession #MSV000097532.

## References

[R1] Benne R, Kasperaitis M, Voorma HO, Ceglarz E, Legocki AB (1980). Initiation factor eIF-2 from wheat germ. Purification, functional comparison to eIF-2 from rabbit reticulocytes and phosphorylation of its subunits.. Eur J Biochem.

[R2] Clarke RD, Ranu RS (1987). Characterization of wheat germ initiation factor eIF-2.. Mol Cell Biochem.

[R3] Goodman JK, Zampronio CG, Jones AME, Hernandez-Fernaud JR (2018). Updates of the In-Gel Digestion Method for Protein Analysis by Mass Spectrometry.. Proteomics.

[R4] Lax SR, Lauer SJ, Browning KS, Ravel JM (1986). Purification and properties of protein synthesis initiation and elongation factors from wheat germ.. Methods Enzymol.

[R5] Liu H, Gong X, Deng H, Tan J, Sun Y, Wang F, Wu W, Zhou Z, Xu R, He H, Lo C (2022). The Rice Aspartyl-tRNA Synthetase YLC3 Regulates Amino Acid Homeostasis and Chloroplast Development Under Low Temperature.. Front Plant Sci.

[R6] Luna E, van Hulten M, Zhang Y, Berkowitz O, López A, Pétriacq P, Sellwood MA, Chen B, Burrell M, van de Meene A, Pieterse CM, Flors V, Ton J (2014). Plant perception of β-aminobutyric acid is mediated by an aspartyl-tRNA synthetase.. Nat Chem Biol.

[R7] McWhite CD, Papoulas O, Drew K, Cox RM, June V, Dong OX, Kwon T, Wan C, Salmi ML, Roux SJ, Browning KS, Chen ZJ, Ronald PC, Marcotte EM (2020). A Pan-plant Protein Complex Map Reveals Deep Conservation and Novel Assemblies.. Cell.

[R8] Merrick WC, Kemper WM, Anderson WF (1975). Purification and characterization of homogeneous initiation factor M2A from rabbit reticulocytes.. J Biol Chem.

[R9] Murphy S, Henry M, Meleady P, Ohlendieck K (2018). Utilization of dried and long-term stored polyacrylamide gels for the advanced proteomic profiling of mitochondrial contact sites from rat liver.. Biol Methods Protoc.

[R10] Schwarzenbacher RE, Luna E, Ton J (2014). The discovery of the BABA receptor: scientific implications and application potential.. Front Plant Sci.

[R11] Schwarzenbacher RE, Wardell G, Stassen J, Guest E, Zhang P, Luna E, Ton J (2020). The IBI1 Receptor of β-Aminobutyric Acid Interacts with VOZ Transcription Factors to Regulate Abscisic Acid Signaling and Callose-Associated Defense.. Mol Plant.

[R12] Spremulli LL, Walthall BJ, Lax SR, Ravel JM (1979). Partial purification of the factors required for the initiation of protein synthesis in wheat germ.. J Biol Chem.

[R13] Treadwell BV, Mauser L, Robinson WG (1979). Initiation factors for protein synthesis from wheat germ.. Methods Enzymol.

[R14] Walthall BJ, Spremulli LL, Lax SR, Ravel JM (1979). Isolation and purification of protein synthesis initiation factors from wheat germ.. Methods Enzymol.

